# Allele-Specific Deletions in Mouse Tumors Identify Fbxw7 as Germline Modifier of Tumor Susceptibility

**DOI:** 10.1371/journal.pone.0031301

**Published:** 2012-02-13

**Authors:** Jesus Perez-Losada, Di Wu, Reyno DelRosario, Allan Balmain, Jian-Hua Mao

**Affiliations:** 1 Helen Diller Family Comprehensive Cancer Center, Cancer Research Institute, University of California San Francisco, San Francisco, California, United States of America; 2 Instituto de Biología Molecular y Celular del Cancer, Consejo Superior de Investigaciones Cientificas/Universidad de Salamanca, Salamanca, Spain; 3 Life Sciences Division, Lawrence Berkeley National Laboratory, Berkeley, California, United States of America; Univesity of Texas Southwestern Medical Center at Dallas, United States of America

## Abstract

Genome-wide association studies (GWAS) have been successful in finding associations between specific genetic variants and cancer susceptibility in human populations. These studies have identified a range of highly statistically significant associations between single nucleotide polymorphisms (SNPs) and susceptibility to development of a range of human tumors. However, the effect of each SNP in isolation is very small, and all of the SNPs combined only account for a relatively minor proportion of the total genetic risk (5–10%). There is therefore a major requirement for alternative routes to the discovery of genetic risk factors for cancer. We have previously shown using mouse models that chromosomal regions harboring susceptibility genes identified by linkage analysis frequently exhibit allele-specific genetic alterations in tumors. We demonstrate here that the *Fbxw7* gene, a commonly mutated gene in a wide range of mouse and human cancers, shows allele-specific deletions in mouse lymphomas and skin tumors. Lymphomas from three different F1 hybrids show 100% allele-specificity in the patterns of allelic loss. Parental alleles from *129/Sv* or *Spretus/Gla* mice are lost in tumors from F1 hybrids with *C57BL/6* animals, due to the presence of a specific non-synonymous coding sequence polymorphism at the N-terminal portion of the gene. A specific genetic test of association between this SNP and lymphoma susceptibility in interspecific backcross mice showed a significant linkage (p = 0.001), but only in animals with a functional *p53* gene. These data therefore identify *Fbxw7* as a *p53*-dependent tumor susceptibility gene. Increased *p53*-dependent tumor susceptibility and allele-specific losses were also seen in a mouse skin model of skin tumor development. We propose that analysis of preferential allelic imbalances in tumors may provide an efficient means of uncovering genetic variants that affect mouse and human tumor susceptibility.

## Introduction

Susceptibility to cancer development is consequence of a combination of genetic and environmental factors. Whereas a wide range of environmental agents may contribute to cancer development, the genetic component of risk is difficult to dissect [1]. Genetic risk factors for cancer are either in the high penetrance category, such as mutations in *BRCA1/2* that cause familial breast and ovarian cancers, or are classified as low penetrance variants that work additively or in complex combinations to increase risk [1], [2]. Relatively little is known about the genes in this latter category, which are nevertheless likely to be very important determinants of the developmental risk of many cancer types [2], [3]. Present approaches to the detection of low penetrance tumor susceptibility alleles in humans involve association studies using DNA samples from hundreds or thousands of cancer patients, and an equal number of well-matched controls. Such studies are plagued by confounding factors such as population heterogeneity, weak effects, and genetic interactions, and require very large numbers of cases and controls to reach statistical significance [4]–[6]. For many complex trait diseases, including cancer, the total number of significant SNP associations detected can only account for a very small proportion of the total genetic risk [7], leading to considerable discussion of the best ways to discover the majority of disease-causing alleles in the human population.

Mouse models offer an important alternative approach to the study of cancer susceptibility. Studies on mice have revealed that tumor predisposition in different strains is controlled by multiple loci that exhibit complex genetic interactions [8], [9]. Importantly, in these mouse crosses only two alleles at each locus are segregating in the population, and the large differences in susceptibility are therefore due to combinations of common alleles. However, the resolution of mapping studies, based on linkage analysis involving inbred strains alone, is poor [1], [3]. Analysis of somatic events in tumors could provide more high resolution information from relatively smaller numbers of samples [10], [11], due to the fact that genetic instability in tumors results in specific genetic aberrations leading to copy number gains or losses of important cancer genes. Indeed, analysis of somatic deletions was instrumental in identification of the causal genes for hereditary cancer syndromes such as Li-Fraumeni syndrome and retinoblastoma. Predisposition to tumor development caused by a germline mutation was accompanied by tumor-specific loss of the wild type allele (*TRP53* and *RB* respectively), thus establishing a precedent for use of somatic genetic alterations for identification of germline tumor susceptibility genes [12], [13]. The information obtained from somatic events in tumors combined with linkage analysis could provide the opportunity to obtain more high resolution information from relatively smaller numbers of samples. Thus, fine mapping of somatic recombination events, deletions and amplifications, together with genotyping to detect preferential allelic imbalance, could offer important clues to the localization of polymorphisms of low penetrance genes that influence cancer susceptibility [10], [11], [14].

We previously identified the *Fbxw7* gene (also known as *hCDC4*, *Fbw7* and *hAGO*) as a tumor suppressor gene for radiation-induced lymphoma [15], [16]. The Fbxw7 gene encodes an F-box protein, essential for the ubiquitination of different oncoproteins, including c-Myc [17], [18], c-Jun [19], cyclin E [20]–[22], different members of the Notch family [23]–[25], Aurora-A [15], [26], and mTor [27], [28]. *FBXW7* gene mutations have been found in cancers from a wide spectrum of human tissues, such as bile duct, the haematopoietic system, bone, brain, breast, colon, endometrium, stomach, lung, ovary, pancreas, prostate, and head and neck [29], [30]. The overall frequency of point mutations in *FBXW7* in human cancers is about 6% [31]. Deletion of the *Fbxw7* gene in mice leads to embryonic lethality, but heterozygous mice develop normally [32], [33]. Although they do not develop spontaneous tumors, radiation exposure gives rise to different types of tumors, including a range of epithelial cancers [15].

Since *Fbxw7* is frequently mutated or deleted in a large proportion of lymphomas from *p53* heterozygous mice [15], we first analyzed the possibility that these deletions may preferentially involve one parental allele. Here we demonstrate that a strain-dependent polymorphism in the N-terminus of Fbxw7 causes 100% selectivity in patterns of alleleic loss in mouse lymphomas. Genetic linkage studies confirmed that this polymorphism confers increased risk of tumor development in a *p53*-dependent manner. Extension of this approach to human samples may identify a proportion of the genetic variants that affect cancer risk, but are not detected by standard linkage of genetic association studies.

## Results

### Allele-specific loss of *Fbxw7* in radiation-induced mouse Lymphomas

**Figure 1:**
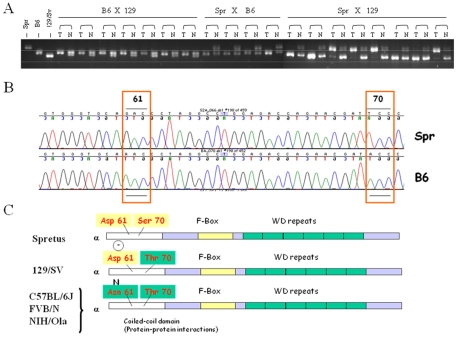
Detection of allele-specific loss of *Fbxw7* gene in radiation-induced thymic lymphomas. (A) PCR analysis at the *Fbxw7* locus: Tumors derived from F1 hybrids between *C57BL/6* and *129/Sv*, or *C57BL/6* and *Mus spretus*, showed consistent loss of the *129/Sv* or *Mus spretus* alleles, respectively; whereas tumors derived from *129/Sv* and *Mus spretus* hybrids showed loss of either *129/Sv* or *Spretus* alleles at the same frequency. (B) Sequence analysis showing the polymorphism in *Fbxw7* between *C57BL/6* (B6) and *Mus spretus* (Spr). (C) Comparison of the amino acid sequences at the *Fbxw7α* polymorphic region in *Mus spretus*, *129/Sv*, *C57BL/6*, *FVB/N*, *NIH/Ola* strain by sequencing.

We have previously proposed that it may be possible to identify germline polymorphisms that influence susceptibility by analysis of allele-specific deletions or amplifications in mouse or human tumors [10], [11]. Thus, we carried out a detailed analysis of the directionality of allele loss of *Fbxw7* in lymphomas from a series of different *p53*+/− F1 hybrid mice. In contrast to tumors derived from *129/Sv×Mus spretus (Spretus/Gla)* hybrids, which showed 50% loss of markers on each parental chromosome 3 (Fig. 1A), tumors derived from F1 hybrids between *C57BL/6* and *Mus spretus*, or between *C57BL/6* and *129/Sv*, showed consistent loss of the *Mus spretus* or *129/Sv* alleles respectively (Fig. 1A). Although these examples of chromosomal imbalance involve large regions of mouse chromosome 3 that include the region containing *Fbxw7*, our previous studies demonstrated that *Fbxw7* was indeed the driver gene for these losses, since tumors exhibited intragenic *Fbxw7* deletions or mutations, and lymphomas from *p53*+/−*Fbxw7*+/− mice did not show any evidence of significant deletions on this chromosome [15]. These data suggested that *129/Sv* and *Spretus/Gla* may carry similar haplotypes in the *Fbxw7* region, while *C57BL/6J* should carry a different sequence. Sequence analysis demonstrated that *Spretus/Gla* and *129/sv* strains share a non-synonymous coding sequence polymorphism at position 61 (GAC) that encodes Aspartic acid whereas in *C57BL/6* mice there is an Asparagine residue (AAC) leading to the disappearance of a negative charge (Fig. 1B). Additional sequencing of other strains showed that this polymorphism segregates in many common laboratory strains of mice (Fig. 1C, Table 1).

**Table 1:** Distribution of *Fbxw7* alleles among different mouse strains by sequencing analysis.

	G allele		A allele	
Strains	Spretus/EiJ	BABL/C	C57BL/6J	RIII/DmM
	Spretus/Gla	AKR/J	SWR	A/WySnJ
	Spretus/Pas	Cast/Ei	CBA/J	NOD/LtJ
	129/Sv		C3H/He	Non/Ltj
	LG/J		A/J	SM/J
	Lp/J		C57BR/cdJ	
	DBA/1J		NIH/O	
	DBA/2J		FVB/N	
	129/REJ		SJL/J	
	PL/J		SenCarC/PtJ	

### Asp61Asn polymorphism in *Fbxw7* is associated with susceptibility to development of radiation-induced lymphoma

**Figure 2:**
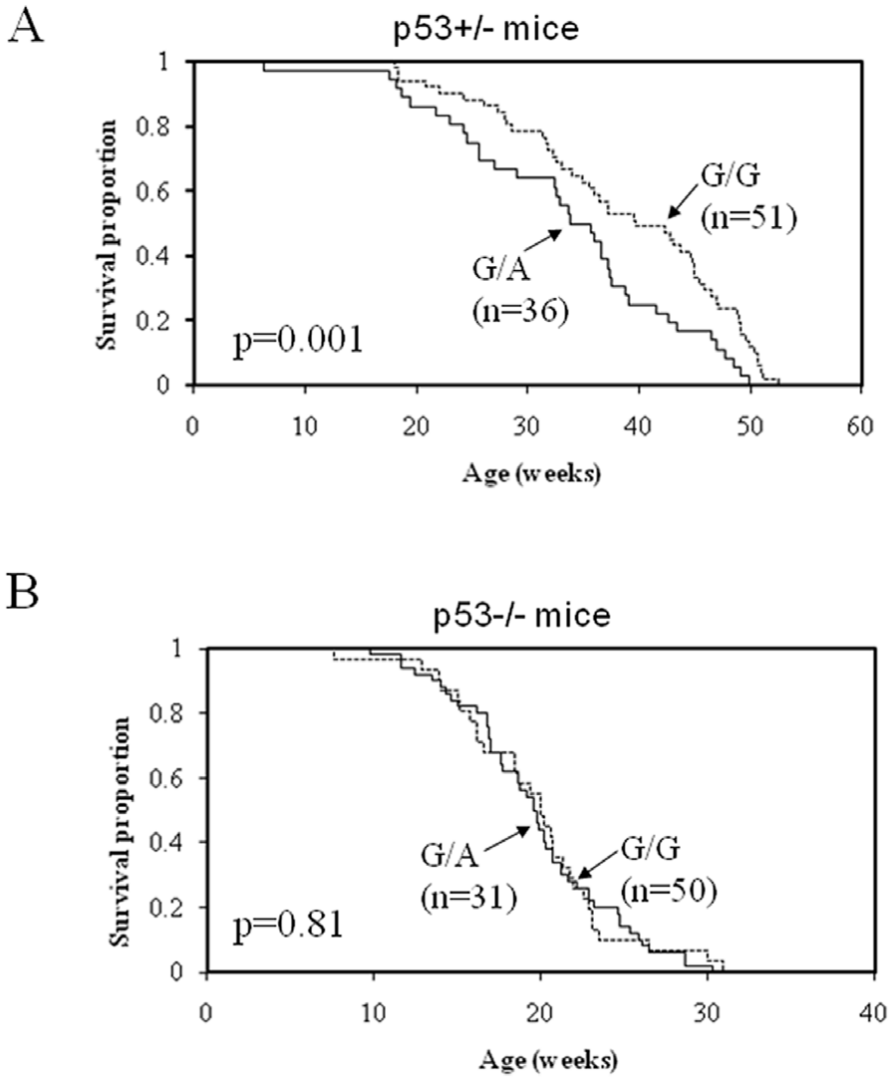
*Fbxw7* is a polymorphic genetic modifier of radiation-induced lymphoma development. Animals from a backcross between *Mus spretus* and *129*×*B6* p53-deficient mice were genotyped at the *Fbxw7* polymorphic locus and monitored for lymphoma development after 4 Gy whole body γ-radiation exposure (A) Lymphoma development in *p53+/−* mice, and (B) in *p53−/−* mice, respectively.

Having found a common allele-specific deletion involving *Fbxw7* on chromosome 3, we then asked whether this polymorphism is associated with altered susceptibility to development of radiation-induced lymphomas. We previously carried out a cross between outbred *Spretus/Gla* and *p53−/−* mice of a mixed *C57BL/6* and *129/Sv* genetic background (129×B6 mice). These interspecific F1 hybrid *p53+/−* animals were backcrossed to the *129/B6 p53*−/− mice to generate a backcross population (n = 168) of which approximately half of the animals were *p53*+/−, while the remainder were homozygous for the *p53* null allele (*p53*−/−) [15]. Since all 3 *Fbxw7* alleles were segregating within this population, we tested the possibility of association between lymphoma development and the presence of the candidate susceptibility SNP in *Fbxw7*. While there was no detectable influence of the Asp61Asn polymorphism in *Fbxw7* on lymphoma development in *p53*−/− mice (Fig. 2B), a significant effect was detected at the same SNP in radiation-induced lymphomas from *p53*+/− mice (Fig. 2A). The result is in agreement with the observation that the tumor suppressor activity of *Fbxw7* is also *p53*-dependent [15], [16]. To our knowledge, this is the first example of a natural genetic variant that influences tumor development in a *p53*-dependent manner.

### *Fbxw7* Participates in Skin Tumor Progression

**Figure 3:**
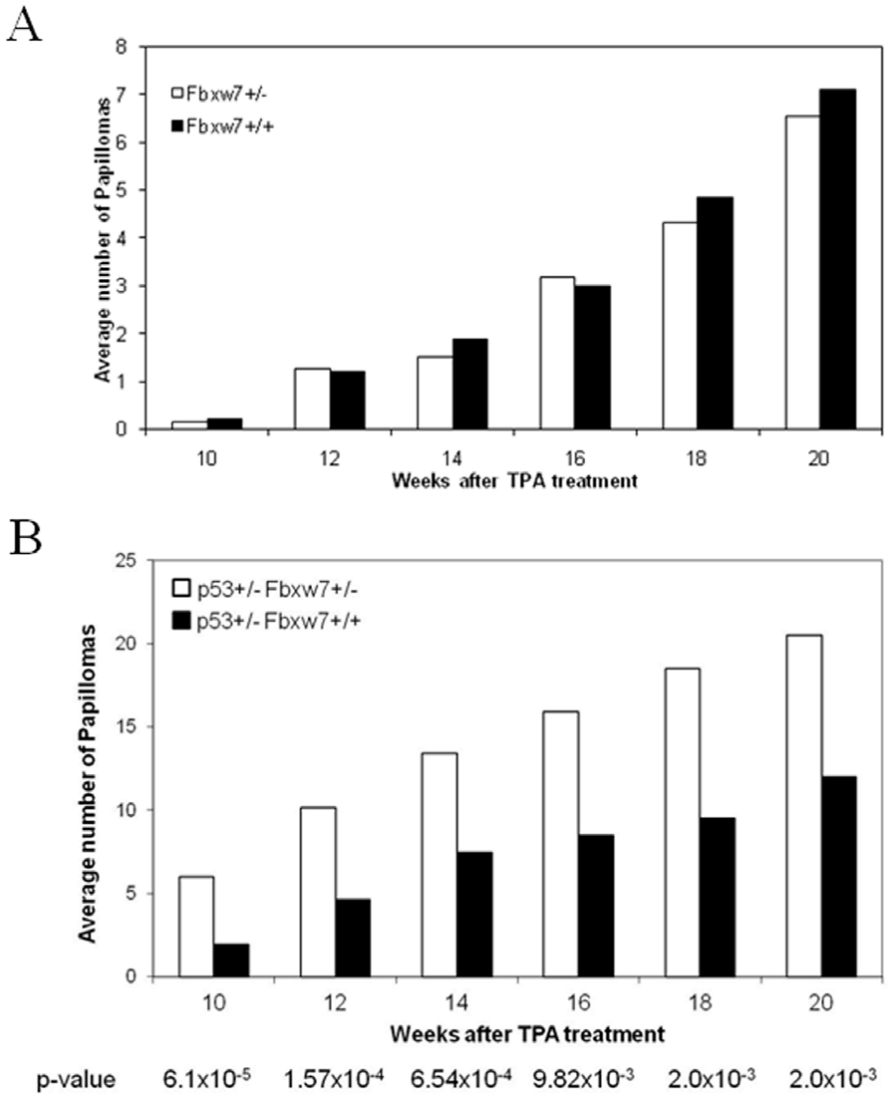
Skin cancer induction by DMBA-TPA protocol in *Fbxw7* deficient mice. (A) Loss of a single copy of *Fbxw7* does not significantly affect papilloma development: Average of papillomas in *Fbxw7+/−* and wild type mice. (B) Loss of *Fbxw7* increases susceptibility to papilloma development in a *p53*-heterozygous background. There were significant differences in the average of papilloma development between *p53+/−Fbxw7+/+* and *p53+/−Fbxw7+/−* mice (Mann-Whitney test).

Loss or mutation of *FBXW7* has been identified in a wide range of human tumors of both mesenchymal and epithelial origin [29]. To clarify the role of *Fbxw7* in epithelial cancer development, we investigated the consequences of *Fbxw7* deficiency in the DMBA/TPA mouse model of skin cancer development. In this model, skin tumors are initiated by treatment with a single dose of the carcinogen DMBA, which induces *H-ras* gene mutations in target cells. Subsequent promotion of these cells by treatment with TPA results in formation of benign papillomas, a subset of which undergoes malignant progression to carcinomas. Since complete loss of *Fbxw7* is lethal in mice, we examined the effects of partial *Fbxw7* deficiency on skin tumor development, both in wild type mice and in animals lacking one functional copy of the *p53* gene. Groups of 19 *Fbxw7*+/+, 28 *Fbxw7*+/−, 32 *p53*+/− and 34 *p53*+/−*Fbxw7*+/− mice were initiated by a single dose of DMBA and promoted twice weekly with TPA. Mice that were wild type at the p53 locus showed no effect of reduced *Fbxw7* gene dosage on papilloma number (Fig. 3A). However there was a strong and statistically significant increase in the number of papillomas in *p53*+/−*Fbxw7*+/− mice compared with *p53*+/− mice (Fig. 3B). We conclude that *Fbxw7* is an important regulator of skin tumor progression, and that a partial germline deficiency causes increased susceptibility to papilloma development, particularly in the context of a reduced gene dosage for *p53*. These data extend and support the conclusion above that *Fbxw7* is an important tumor suppressor gene that interacts strongly with the p53 pathway.

**Figure 4:**
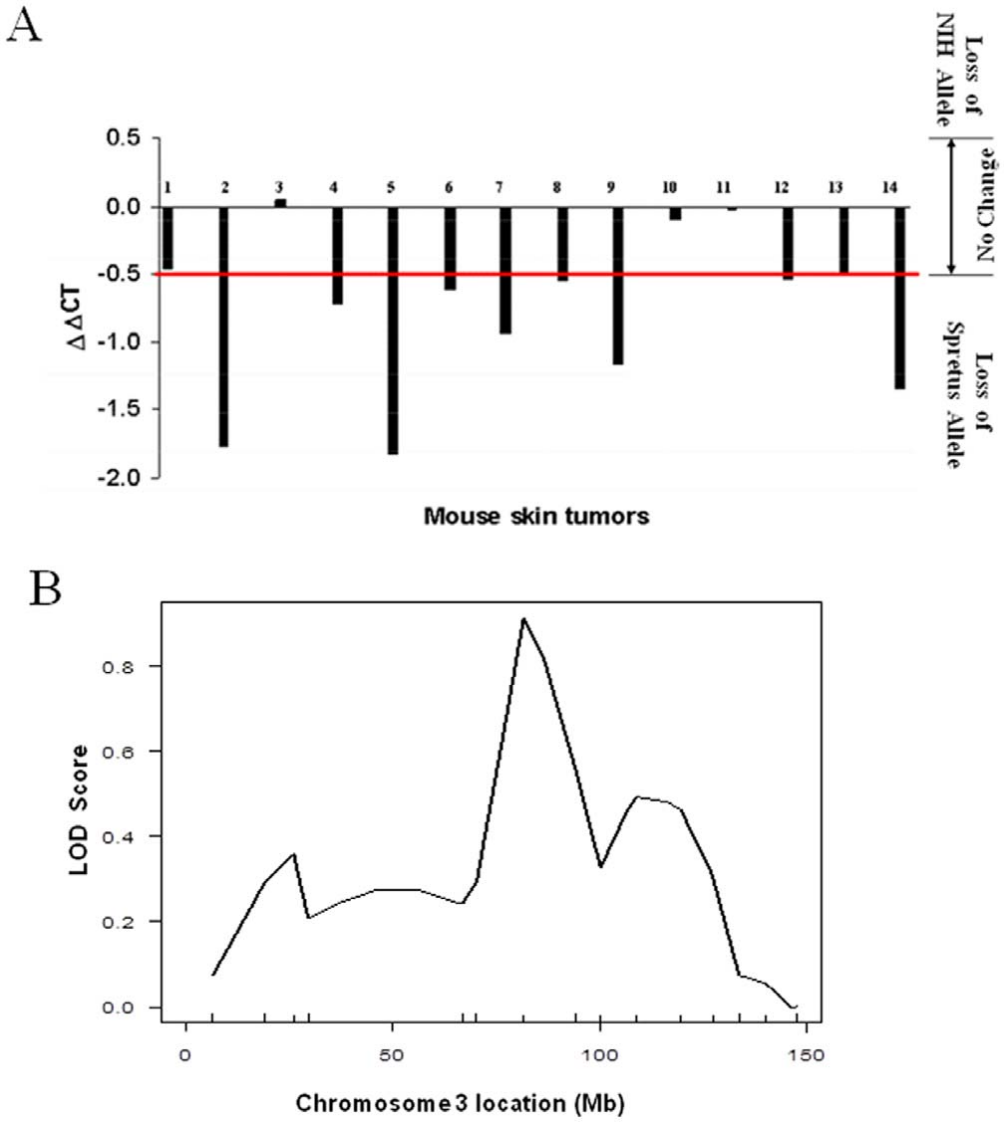
Allele-specific change and linkage analysis of Fbxw7 locus in skin tumors. (A) Analysis of allele-specific *Fbxw7* gene copy number by QPCR in primary skin tumors. The *Fbxw7* allele from the *Mus spretus* parent was deleted in early stages of skin cancer development. Samples 1, 6 and 7 are papillomas; samples 13 and 14 are spindle carcinomas; all the rest are squamous carcinomas. ΔΔCT less than −0.5 indicates loss of the *Mus Spretus* allele. (B) LOD scores for number of papillomas on Chromosome 3 were generated by R/QTL.

We next investigated the possibility that allele-specific deletions in Fbxw7 locus may occur in skin tumors. Due to normal tissue contamination, it is hard to detect allele-specific change in skin tumors by regular PCR described in lymphoma studies. Thus we designed TaqMan probes specific for Fbxw7 polymorphisms discovered above. Gene copy number changes at the *Fbxw7* locus were examined by quantitative PCR (TaqMan) analysis of skin tumors from interspecific backcross mice derived by crossing male *Spretus/Gla* with female mice of the *NIH/O* strain that carry the G and A alleles respectively (see Table 1). Female F1 hybrid mice generated from this cross were backcrossed to the parental *NIH/O* strain to generate a backcross population (NIHBX) as previously described [34], [35]. *Fbxw7* deletions were detected in some papillomas and in the majority of the carcinomas studied (Fig. 4A). Analysis of the pattern of allele-specific losses in these tumors indicated that the preference was overwhelmingly for loss of the *Fbxw7* allele inherited from the *Mus spretus* parent. These data indicate that loss of *Fbxw7* is an important event in skin cancer progression, and demonstrate that the strain-specific polymorphism affects the directionality of the deletion in the same manner as was seen for lymphomas.

In the lymphoma model described above, the presence of the G/A allele was linked to the allele-specific deletions and also, by SNP association analysis, to susceptibility to lymphoma development. We investigated the possibility that allele-specific losses of *Fbxw7* in the NIHBX population may also predict linkage to a skin tumor susceptibility locus in the region containing the *Fbxw7* gene on chromosome 3. Interestingly, although allele-specific deletions were clearly seen in tumors from this backcross, no obvious linkage was detected to markers near the *Fbxw7* locus (Fig. 4B). We conclude that in this skin model, analysis of preferential allelic imbalance in tumors leads to detection of a germline susceptibility locus that is not detected by standard linkage analysis.

## Discussion

The identification of low penetrance cancer susceptibility genes is a major challenge, and fraught with difficulties in standard human population studies. In this work we have established evidence that supports the approach of studying somatic mutation events within the tumors, in combination with linkage analysis, to identify polymorphic allelic variants that have cell-autonomous effects on tumor development. Analysis of allele-specific deletions of *Fbxw7* locus in mouse lymphomas showed that the pattern in three different sets of F1 hybrid was 100% specific: the allele contributed by the *C57BL/6* parent was selectively retained, while alleles from either Mus spretus or *129/Sv* were preferentially lost, in lymphomas from the respective F1 hybrid animals. An association study in a backcross in which all 3 alleles were segregating confirmed that the AAC (Asn) allele inherited from the *C57BL/6* parent conferred greater risk of lymphoma than the GAC (Asp) allele from either *129/Sv* or *Mus spretus*. An important conclusion is that while the genetic effect of the polymorphism on overall cancer risk is small and this variant is clearly in the “low penetrance’ category, the effect shows 100% penetrance in the pattern of allele-specific loss of heterozygosity in lymphomas. Application of this approach to human cancers may therefore identify genetic variants that only have very small and possibly undetectable effects on cancer risk in GWAS, but have strong effects on somatic genetic changes.

Our results on the skin tumor model lend strong support to the conclusion that *Fbxw7* variants contribute to cancer risk. In this model, loss of heterozygosity at the *Fbxw7* gene is specific for the Spretus allele, and no examples were seen of loss of the parental *NIH/O* (AAC) allele. In addition, germline deletion of one copy of *Fbxw7* clearly increased skin tumor susceptibility, particularly in the context of *p53* deletion. Nevertheless, in the same backcross population in which the allele-specific losses were seen, no evidence was detected of a significant germline skin tumor susceptibility gene at this locus using a range of markers on mouse chromosome 3. The reasons for this observation are unclear, but it is possible that additional germline susceptibility or resistance alleles for skin cancer map within the same linkage region as *Fbxw7*. If these act in the opposite direction, the effect due to susceptibility through the *Fbxw7* AAC allele may be masked by the opposing genetic effects of these linked variants, as discussed previously [1]. The results of linkage analysis may therefore depend on the tissue and tumor type under investigation, where in some cases closely linked genetic variants that act in opposite directions may confound detection of susceptibility alleles. Similar tissue-specific effects may underlie the differential effect of *p53* status on germline deletion of *Fbxw7*.

It has been reported that monoallelic deletion of *Fbxw7* in different tumor systems, in human and mouse, is a frequent event during cancer progression [15], [29], and that *Fbxw7* acts as a haploinsufficient tumor suppressor gene[15], [29]. In the light of these results on mouse models of cancer, it is possible that specific genetic variants in human *FBXW7*, or indeed of other common human tumor suppressors, may also affect the patterns of allelic losses in tumors. Such changes may be more difficult to detect in human tumors than are allele-specific gene amplifications [10] which occur over a wider range of copy number changes and are less subject to confounding by the presence of contaminating normals cell that increase the signal-noise ratio. Nevertheless, tumor microdissection in combination with genome-wide analysis of allelic imbalances may reveal new opportunities to detect germline variants that influence cancer risk.

In preliminary studies we have been unable to detect any effect of the specific *C57BL/6* or *Mus spretus* alleles on intracellular localization of epitope tagged Fbxw7 proteins or any reproducible differences in protein stability (data not shown). While a human non-synonymous coding sequence polymorphism has been found in the same region of the human gene, this is only seen at low frequency in populations of African descent (rs6842544: AGA→GGA) (http://www.ncbi.nlm.nih.gov/snp). Further studies will be required to investigate possible allele-specific loss or retention of this allele in tumors from heterozygous patients. Elucidation of the mechanisms by which this and other *Fbxw7* genetic variants act may allow us in future studies to recognize patients who are at high risk of cancer development, or who may be more likely to respond to therapies targeted at this pathway. The recent identification of mTOR as a downstream target of Fbxw7 [27], [28] suggests that germline or somatic changes may affect tumor responses to known inhibitors of the mTOR pathway such as rapamycin, and such investigations are presently in progress in this laboratory.

## Materials and Methods

### Mouse breeding and tumor induction

#### Skin Cancer

The mice used for this study have been described previously for skin tumor susceptibility [11], [34], [35]; in short, female interspecific F1 hybrid mice between *NIH/Ola* and *Spretus/Gla* were crossed with male *NIH*/Ola to generate the F1 backcross mice (*NIH/Ola×Spretus/Gla*)×NIH/Ola. Additionally, *Fbxw7+/−* knockout mice, generated at Dr. Nakayama's laboratory [32], were crossed with P53 knockout heterozygous mice. *P53* and *Fbxw7* single- or double-heterozygous knockout mice were generated by crossing *Fbxw7*+/− with p53+/− mice. The F1 backcross mice, together with wild type, *Fbxw7+/−*, *P53+/−* and double heterozygous *Fbxw7+/−P53+/−* mice were treated with DMBA and 12-*O*-tetradecanoylphorbol-13-acetate according to the standard two-stage carcinogenesis protocol [34].

#### Radiation-induced Lymphoma

This cohort of mice was described before for radiation-induced induced lymphoma susceptibility [15], [36]. Briefly, F1 hybrid mice were generated by crossing females *p53−/− 129/B6* with males p53 wild type *Spretus/Gla*, and F_1_ backcross mice were obtained by mating the female F_1_ hybrids with male *p53−/− 129/B6* mice. The 5-week-old *129/Sv*, F_1_ and F_1_ backcross mice were exposed to a single dose of 4 Gy whole-body irradiation and observed daily until they were sick, then sacrificed and autopsied.

Mice were bred and treated under University of California at San Francisco (UCSF) Laboratory Animal Resource Center (LARC) regulations.

### Allele specific LOH determination

#### PCR Analysis

For DNA preparation, tumors were ground into a fine powder in liquid nitrogen. DNA was isolated using standard phenol/chloroform extraction following over-night 55°C incubation with Proteinase-K. All tumor DNA samples were examined together with normal DNA from the same mice, to take account of variation in *Fbxw7* alleles. Tumor and corresponding normal DNA was used for LOH studies with microsatellite markers at the *Fbxw7* locus [15]. PCR amplification was performed in 20-µL volume containing 1× PCR buffer (Bioline), 200 µM of each dNTP (Pharmacia), 6.6 µM of each primer (Qiagen Operon), 1.5 mM MgCl2, 2 units of Taq polymerase (Bioline), and 80 ng of DNA. Amplifications were initially denatured for 3 min at 94°C, followed by 35 cycles of 30 sec at 94°C, 30 sec at 55°C or 52°C, and 30 sec at 72°C. PCR products were electrophoresed in 4% (3% NuSieve/1% normal) agarose gel and visualized by ethidium bromide staining.

#### Quantitative Real Time PCR (QPCR) Analysis

Primers and TaqMan probes were designed using the ABI Primer Express Software (version 1.5). The absence of non-specific amplification was confirmed by analyzing the PCR amplification by 3% agarose gel electrophoresis and ethidium bromide staining. The sequences were as follows: Fbxw7 forward primer: GCGCGGAATGGTGAACTT; Fbxw7 reverse primer: CGTTCTGGTCTCCAGGCCT; probe for Fbxw7-GAC allele: 5′-FAM- TGGGTGCAGACCCTA-MGB-3′; probe for Fbxw7-AAC allele: 5′-VIC- TGGGTGCAAACCCTA -MGB-3′. The probe for the detection of amplified products was labeled with 6-carboxifluorescein (FAM) in the 5′end, all the reporters were quenched by 6-carboxy-tetramethyl-rhodamine (TAMRA) conjugated at the 3′end. All probes were purchased in Applied Biosystems. PCRs for each sample were carried out in triplicate and repeated at least twice in 50 uL volume consisting in: 25 uL TaqMan 2× PCR Mater Mix (Applied Biosystems), 100 nM TaqMan probe, forward and reverse primers (100 uM). A master mix of the components including the equivalent of 125 ng of DNA per well (5 uL at 25 ng/uL) was made and aliquoted into a 96-well optical plate (Applied Biosystems). PCRs were performed according to the thermal profile: 1 cycle 50°C 2 min, 1 cycle 95°C 10 min, 40 cycles 95°C 15 sec and 60°C 1 min. For quantification of gene relative copy number, we used comparative Ct method [37], [38]. The Ct values for each set of triplicates were averaged. For *Fbxw7* copy number |ΔΔCt|>0.5 were considered to be losses.

### Statistical and linkage analysis

The Kaplan–Meier method was used to compare the tumor development after irradiation of mice carrying different alleles of Fbxw7, and the Mann-Whitney test for comparing number of papillomas between different genotypes of mice. Statistical analysis was performed using SPSS version 12.0 (SPSS, Chicago, IL). Linkage analysis was carried out using R/QTL.

### Ethics statement

The animal protocols were approved by University of California at San Francisco (UCSF) Laboratory Animal Resource Center (LARC) (AN084982).
